# Barriers and facilitators to exclusive breastfeeding among formally employed mothers in urban Indonesia

**DOI:** 10.1186/s12889-025-25214-6

**Published:** 2025-11-12

**Authors:** Ni Putu Gita Prastita, Deni Kurniadi Sunjaya, Hanom Husni Syam, Sofie Rifayani Krisnadi, Dzulfikar Djalil Lukmanul Hakim, Ruswana Anwar, Hadi Susiarno

**Affiliations:** 1 Faculty of Medicine, Universitas Padjadjaran, Bandung, Indonesia; 2 Department of Public Health, Faculty of Medicine, Universitas Padjadjaran, Bandung, Indonesia; 3 Department of Obstetrics and Gynecology, Faculty of Medicine, University Padjajaran, Hasan Sadikin Hospital Bandung, Bandung, West Java Indonesia; 4 Department of Pediatrics, Faculty of Medicine, adjadjaran University, Hasan Sadikin Hospital Bandung, Bandung, West Java Indonesia

**Keywords:** Exclusive breastfeeding, Working mothers, Barriers, Facilitators, Qualitative research, Indonesia, Lactation support

## Abstract

**Background:**

Despite global and national recommendations, exclusive breastfeeding (EBF) remains suboptimal among formally employed mothers in Indonesia. Although legal protections exist, real-world barriers persist that complicate the breastfeeding journey for working women. This study explored factors barriers or facilitators to exclusive breastfeeding among formally employed mothers in urban Indonesia.

**Methods:**

A qualitative exploratory design was employed using a constructivist paradigm to explore the experiences of formally employed mothers in sustaining exclusive breastfeeding while working. A total of 12 participants were interviewed through theoretical sampling. They were aged 25–36 years and had infants aged 6–12 months. Data collection was conducted via in-depth semi-structured interviews held online. The interviews were analyzed using inductive thematic analysis with the aid of ScreenQ (qualitative analysis software), which generated 131 initial codes, 23 categories, 7 subthemes, and two overarching themes: internal and external factors influencing breastfeeding continuation.

**Results:**

Internal facilitators included maternal knowledge, self-efficacy, and adaptive breastfeeding practices, while internal barriers involved fatigue, self-doubt, and difficulty translating knowledge into practice. External facilitators encompassed partner and coworker support, flexible workplace policies, and lactation-friendly facilities. Conversely, barriers included inflexible schedules, short maternity leave, stigma, and inconsistent caregiving.

**Conclusions:**

Exclusive breastfeeding (EBF) among working mothers is shaped by complex interactions between individual strengths and structural conditions. Supportive policies and environments are essential to help bridge the gap between mothers’ intentions and practical realities. Implementation of enforceable workplace protections and extension of maternity leave are essential to sustain EBF in Indonesia. It is equally important to establish employer accountability mechanisms—such as government monitoring and enforcement of workplace lactation rooms and breastfeeding breaks—that can be implemented in the short term.

**Supplementary Information:**

The online version contains supplementary material available at 10.1186/s12889-025-25214-6.

## Key Messages

• **What is already known:**

Exclusive breastfeeding rates are lower among working mothers due to occupational andstructural challenges.

• **What this paper adds:**

This study identifies both internal (e.g., self-efficacy, knowledge) and external (e.g., workplacesupport, caregiver reliability) factors that act as barriers or facilitators to EBF among formallyemployed mothers in Indonesia.

• **Implications for practice and policy:**

Workplace accommodations, extended maternity leave, consistent caregiver training, andculturally sensitive breastfeeding education are essential to sustain EBF in working mothers.

Stakeholders must prioritize breastfeeding as a collective societal responsibility, not just amaternal task.

**Supplementary Information:**

The online version contains supplementary material available at 10.1186/s12889-025-25214-6.

## Introduction

Exclusive breastfeeding (EBF) is globally recognized as essential for infant and maternal health. The World Health Organization (WHO) recommends that infants be exclusively breastfed for the first six months of life, receiving only breast milk—either directly, expressed, or from a wet nurse—without any additional food or drink, except for prescribed medications, vitamins, or minerals [1]. Breastmilk provides critical nutrients, hormones, and bioactive compounds that support optimal growth, development, and immune protection. EBF has been shown to reduce the risk of obesity, allergies, diabetes, and asthma in infants, while also improving cognitive, emotional, and behavioral outcomes [2, 3]. For mothers, it facilitates postpartum recovery and reduces the risk of breast and ovarian cancers, as well as type 2 diabetes[4, 5].

Despite these well-established benefits, exclusive breastfeeding rates in Indonesia remain below national and global targets. According to the 2023 National Health Survey, only 55.5% of infants under six months were exclusively breastfed. Industrialized provinces such as Jakarta, West Java, and Banten report even lower rates. In Bekasi City, for example, only 47.03% of infants received exclusive breastfeeding in 2020 [6]. One major contributing factor is the large number of women in formal employment. The Central Bureau of Statistics (2022) notes that 52.74 million Indonesian women are employed, with 34.33% working in the formal sector. Although formal employment offers structured schedules and legal protections, it often limits the flexibility and environmental support needed to sustain exclusive breastfeeding [7]. In Indonesia, maternity leave is typically limited to three months—1.5 months before and 1.5 months after childbirth—falling short of the six-month exclusive breastfeeding duration recommended by global health authorities[8].

To address this, Indonesia has enacted several regulations to protect and support breastfeeding mothers in the workplace, including Ministerial Decree No. 48/2008, Health Ministry Regulation No. 15/2013, and Law No. 17/2023 supported by Government Regulation No. 28/2024. These laws mandate workplace lactation rooms, nursing breaks, and breastfeeding-friendly policies. Although these regulations apply at the national level, enforcement and implementation vary significantly across provinces and sectors. While some provincial governments and large companies have adopted stronger compliance mechanisms, many workplaces—especially in smaller enterprises—treat these requirements as optional, resulting in wide disparities in mothers’ experiences[9–11]. Globally, the International Labour Organization (ILO) Convention No. 183 recommends at least 14 weeks of paid maternity leave and the right to breastfeeding breaks, while WHO’s NetCode monitoring framework emphasizes workplace environments as critical determinants of breastfeeding success. Recent WHO and UNICEF reports also highlight the necessity of enforceable maternity protection and lactation-friendly workplaces to sustain exclusive breastfeeding in line with global targets [12]. 

Few qualitative studies have examined the lived experiences of formally employed mothers in Indonesia, despite abundant quantitative evidence. This qualitative study aims to fill that gap by exploring how internal factors (e.g., knowledge, self-efficacy) and external factors (e.g., family support, workplace environment) interact to support or hinder exclusive breastfeeding among working mothers in urban Indonesia. By highlighting real-world challenges and facilitators, this research seeks to inform more responsive workplace policies and targeted interventions.

## Materials and methods

This study employed a qualitative exploratory design grounded in the constructivist paradigm, which views knowledge as being co-constructed between researchers and participants. This paradigm emphasizes understanding the lived experiences and subjective interpretations of participants within their social and cultural contexts. The constructivist approach enabled the researcher to deeply interpret how formally employed mothers experience, navigate, and assign meaning to the process of maintaining exclusive breastfeeding while working.

Participants were recruited using theoretical sampling, allowing for concurrent data collection and analysis to inform subsequent recruitment based on emerging concepts. Sampling continued until theoretical saturation was reached. The study population consisted of formally employed mothers from the client database of Mamina Lactation Counselors. Inclusion criteria included mothers aged 21–45 years, with infants aged 6–12 months, who were breastfeeding or had attempted exclusive breastfeeding after returning to work, and who had access to a lactation room or milk expression space at their workplace. Exclusion criteria included full-time work-from-home mothers, those with medical contraindications to breastfeeding, and those whose infants required early formula feeding due to health conditions. Limiting to workplaces with lactation rooms may bias findings toward more supportive contexts.

Data were collected through in-depth online interviews conducted via Zoom, each lasting between 40 and 70 min. The interview guide used in this study was developed by the authors specifically for this research. The interview guide used in this study was developed by the authors and is available in Supplementary File 1. At the beginning of each interview, the interviewer clarified the WHO definition of exclusive breastfeeding to ensure shared understanding. Probing questions were also used to confirm feeding practices. All interviews were audio-recorded with participant consent and transcribed verbatim, resulting in a total of 42 pages of transcripts. All interviews were conducted in Bahasa Indonesia.

Thematic analysis was conducted inductively in four stages: data reduction, open coding, categorization, and theme development. The process was supported by ScreenQ, a qualitative software based on the CASDAQ framework, which facilitated the organization and tracking of codes and categories. The analysis yielded 131 initial codes, grouped into 23 categories, 7 subthemes, and two major themes: internal factors and external factors influencing exclusive breastfeeding. Rigor in this qualitative study was ensured through several strategies. First, credibility was strengthened by source triangulation involving lactation counselors and representatives from the Indonesian Breastfeeding Mothers Association (AIMI) to validate emerging themes. Second, the first author maintained reflexive memos to document assumptions and minimize bias during analysis. Finally, findings and coding decisions were regularly discussed with academic supervisors, serving as peer debriefing to enhance dependability and confirmability.

This study received ethical approval from the Health Research Ethics Committee of Universitas Padjadjaran, Indonesia (Approval 226/UN6.KEP/EC/2025). Written informed consent was obtained from all participants prior to the interviews, and confidentiality and anonymity were maintained throughout the research process.

## Result

Twelve formally employed mothers participated in this study, ranging in age from 25 to 36 years, with infants aged 6 to 12 months. Participants represented a range of professions—including private employees, civil servants, teachers, midwives, and nurses—and were predominantly university graduates. All lived and worked in major urban centers across Java, such as Jakarta, Bekasi, Semarang, Malang, and Bandung, reflecting diverse yet comparably structured work environments. Most participants were primiparous mothers, with only two respondents having two children. Multiparous mothers generally reported greater confidence and practical strategies for maintaining exclusive breastfeeding compared to primiparous mothers, who more frequently expressed concerns and required additional support.

At the time of the interview, 10 of the 12 mothers in Table 1 had maintained exclusive breastfeeding (EBF) for six months, while one had reached five months, and another initiated EBF only upon returning to work. The nature of participants’ jobs appeared to influence their breastfeeding experience. Frontline workers, such as healthcare professionals and teachers, faced more challenges due to rigid schedules and high mobility. In contrast, mothers in office-based roles reported greater flexibility and more consistent access to lactation spaces. These contextual differences underscored the importance of occupational structure in shaping breastfeeding outcomes among working mothers.

**Table 1:** Respondent characteristics

Respondent	Age (Years)	Child’s Age (months)	Occupation	Highest Education	Parity	Duration of maternity leave	Duration of Exclusive Breastfeeding
R01	31	6	Private Employee	Bachelor’s Degree	1	3 months after childbirth	Exclusive breastfeeding for 6 months and ongoing
R02	36	8	Private Employee	Bachelor’s Degree	1	1.5 months before childbirth and 1.5 months after childbirth (total 3 months)	Exclusive breastfeeding for 6 months and ongoing
R03	28	11	Civil Servant	Bachelor’s Degree	1	3 months after childbirth	Exclusive breastfeeding for 6 months and ongoing
R04	31	8	Civil Servant	Bachelor’s Degree	1	3 months after childbirth	Exclusive breastfeeding for 6 months and ongoing
R05	34	7	Midwife	Bachelor’s Degree	1	3 months after childbirth	Exclusive breastfeeding for 6 months and ongoing
R06	26	7	Civil Servant	Diploma	1	3 months after childbirth	Exclusive breastfeeding for 5 months
R07	33	7	Private Employee	Bachelor’s Degree	1	3 months after childbirth	Exclusive breastfeeding for 6 months and ongoing
R08	30	6	Private Employee	Bachelor’s Degree	1	3 months after childbirth	Exclusive breastfeeding for 6 months and ongoing
R09	34	7	Teacher	Bachelor’s Degree	2	3 months after childbirth	Exclusive breastfeeding for 6 months and ongoing
R10	29	6	Private Employee	Bachelor’s Degree	1	3 months after childbirth	Started exclusive breastfeeding at 3 months and ongoing
R11	33	11	Midwife	Diploma (D3)	2	1 months before childbirth and 2 months after childbirth (total 3 months)	Exclusive breastfeeding for 6 months and ongoing
R12	25	12	Nurse	Diploma (D3)	1	3 months after childbirth	Exclusive breastfeeding for 6 months and ongoing

### Perceived barriers and facilitators to exclusive breastfeeding

This study identified two major themes—internal and external factors—that functioned either as barriers or facilitators in sustaining exclusive breastfeeding among formally employed mothers. These are summarized in two sections below.

1. Internal Factors Influencing Exclusive Breastfeeding Among Working Mothers
  This study identified four internal factors that influenced exclusive breastfeeding among formally employed mothers: knowledge, self-efficacy, breastfeeding practices, and internal challenges. Together, these factors shaped the mothers’ capacity to initiate and sustain breastfeeding in the face of professional responsibilities.
  a Aspects and the Role of Knowledge
    Mothers generally understood the benefits of breastfeeding, particularly for their infants’ immunity and emotional development. One mother stated, "Breast milk contains the best nutrients, even better than any expensive formula" (R08), underscoring the perceived superiority of breast milk. However, knowledge about maternal health benefits was less common. Some respondents actively sought information through breastfeeding classes, consultations with lactation counselors, or social media platforms, reflecting a proactive approach. Yet, several mothers found it challenging to apply theoretical knowledge to real-life situations, indicating a persistent gap between understanding and practice.
  b Self-Efficacy
    Self-efficacy refers to mothers’ confidence in their ability to sustain exclusive breastfeeding despite returning to work. Self-efficacy captures the psychological belief that they were capable of continuing exclusive breastfeeding (EBF), rather than the practical barriers encountered. Mothers with strong self-efficacy were more determined to breastfeed despite obstacles such as fatigue, limited facilities, and social stigma. Prior experiences, technological support like hands-free pumps, and peer encouragement enhanced their confidence. These mothers expressed a strong sense of purpose and personal pride in their breastfeeding journey. As one participant shared, "It’s totally worth it—I feel proud, like I’ve achieved something" (R01). Their resilience was evident in how they managed unexpected difficulties, such as needing to pump in cars or workspaces without privacy.
    Some mothers expressed their confidence in sustaining exclusive breastfeeding by framing it as a moral obligation and the child’s inherent right. As one participant shared: “Because I believe that breastfeeding is truly the child’s right.” (R10). This belief strengthened their motivation and sense of responsibility, reflecting a psychological commitment that enhanced their self-efficacy in continuing exclusive breastfeeding.
  c Breastfeeding Practices
    Breastfeeding practices evolved through stages—from the postpartum period, through preparation for returning to work, to daily routines as working mothers. Early adjustments involved coping with exhaustion and new responsibilities. Upon re-entering the workplace, mothers developed personalized strategies such as building a milk supply in advance and balancing direct breastfeeding with pumping. Many had to navigate logistical issues related to storage and space, often adapting available resources to their needs. This flexibility was crucial in maintaining consistency. One mother explained, "I do direct breastfeeding at home and pump at the office—it’s balanced" (R02).
  d Internal Challenges
    In contrast, internal challenges describe the practical and emotional difficulties mothers faced in daily life, such as fatigue, stress, and time constraints, which were distinct from their underlying confidence or motivation. Some felt emotionally overwhelmed, especially when milk supply declined. Others reported discouraging remarks from family or coworkers that compounded their stress. Nonetheless, a common thread among the participants was persistence—rooted in a sense of duty and love—that helped them continue breastfeeding despite internal struggles.
    These findings demonstrate that internal strengths, particularly self-efficacy and practical knowledge, play a central role in exclusive breastfeeding. However, real-world barriers often limit the translation of these strengths into sustainable practice. Bridging this gap requires supportive environments and practical interventions tailored to the lived experiences of working mothers.
2. External Factors Influencing Exclusive Breastfeeding Among Working Mothers
  External factors also played a critical role in either supporting or hindering mothers’ ability to continue exclusive breastfeeding. These included social support systems, workplace accommodations, and broader cultural or logistical challenges.
  a Social Support
    Support from spouses, family members, caregivers, and colleagues significantly enhanced mothers’ confidence and ability to breastfeed. Husbands who actively participated in childcare and household duties helped alleviate mothers’ stress and time burdens. Parental support—such as preparing nutritious meals and avoiding unsolicited advice—created a more positive breastfeeding environment. Domestic helpers, especially those trained in handling expressed milk, were essential in day-to-day caregiving during work hours. Domestic helpers in this study referred to both paid babysitters/household assistants and extended family members such as grandmothers. Their role was crucial due to short maternity leaves. Peer networks, particularly among colleagues who were also breastfeeding, created informal systems of solidarity and encouragement, reducing the sense of isolation. Healthcare professionals and lactation counselors also played a role, offering reliable information and reassurance. As one mother put it, "Sometimes you just want to hear reassurance from an expert" (R03).
  b Workplace Factors
    Workplace infrastructure and policies had a direct impact on breastfeeding outcomes. Some workplaces offered designated lactation rooms equipped with fridges and comfortable seating, which mothers found helpful. However, many mothers had to make do with less ideal spaces, such as prayer rooms or meeting rooms. Inconsistent availability and poor maintenance of lactation spaces often made expressing milk stressful. Few workplaces offered structured pumping breaks, forcing mothers to adapt their own schedules around meetings and work tasks, often at the expense of their health or milk supply. Maternity leave policies were also insufficient, typically capped at three months and divided pre- and post-delivery, which limited the ability to establish and maintain exclusive breastfeeding. One mother noted, "One and a half months before and after birth—too short to complete exclusive breastfeeding" (R02), “Ideally, it would be best if maternity leave could last for 6 months, so that the child can complete exclusive breastfeeding for 6 months. That’s really what I hope for.” (R12).
  c External Challenges
    Cultural perceptions and stigmatization also affected breastfeeding experiences. Some mothers reported being criticized or misunderstood by colleagues, including assumptions that pumping was a way to avoid work. This stigma, often coming from other women, contributed to feelings of discomfort and inadequacy. Inadequate public or workplace facilities further complicated matters, especially during field assignments. Additionally, unreliable childcare arrangements caused anxiety, particularly when caregivers failed to follow milk-feeding schedules or mishandled stored milk. One mother recalled, "I found leftover milk, and she lied about it. I was afraid to switch caregivers" (R08).
    Collectively, these external factors demonstrate how essential a supportive environment is for sustaining exclusive breastfeeding. The availability of social support and breastfeeding-friendly workplace policies can serve as critical facilitators, while inflexible schedules, poor infrastructure, and cultural stigma act as significant barriers. Tailored policies and workplace adaptations, combined with community-based support systems, are necessary to enable working mothers to fulfill their breastfeeding goals.
    To synthesize the findings across the various dimensions explored, the identified internal and external factors influencing exclusive breastfeeding were further categorized into barriers and facilitators. This categorization helps illustrate how certain conditions—such as workplace accommodations, partner support, or personal motivation—acted as enablers, while others—such as fatigue, stigma, or lack of infrastructure—posed significant challenges. The summary presented in Table 2 below consolidates the key barriers and facilitators that shaped the breastfeeding experiences of formally employed mothers in this study.

**Table 2:** Summary of barriers and facilitators to exclusive breastfeeding among y employed mothers

Thematic Area	Facilitators	Barriers	
Knowledge	Awareness of infant health benefits	Limited awareness of maternal health benefits	
	Active learning via classes, social media, lactation counselors	Difficulty applying theory into practice	
Self-Efficacy	High confidence and motivation	Feelings of doubt and discouragement	
	Learned strategies from prior experience – Use of supportive tools (hands-free pumps)	Lack of confidence in early stages	
Breastfeeding Practices	Early planning for work transition	Disrupted routines due to busy schedules	
	Combination of direct breastfeeding and pumping	Limited access to proper lactation facilities	
	Caregiver training		
Internal Challenges	Personal commitment to continue breastfeeding	Sleep deprivation	
	Emotional regulation strategies	Emotional fatigue	
		Physical pain (engorgement, sore nipples)	
Social Support	Supportive spouses, family, and trained caregivers	Inconsistent caregiver practices	
	Encouraging coworkers	Lack of emotional support from close social circle	
	Guidance from healthcare professionals		
Workplace Factors	Availability of designated lactation rooms (in some cases)	No formal pumping breaks	
	Supervisor flexibility and understanding	Limited or poorly maintained lactation spaces	
		Short maternity leave	
External Challenges	Breastfeeding peers who normalize practice	Workplace stigma (especially from female colleagues)	
	Supportive public campaigns (potential)	Inconvenient pumping locations (cars, prayer rooms)	
		Difficulty replacing caregivers	

## Discussion

This study explores how internal and external factors interact to support or hinder exclusive breastfeeding among formally employed mothers in urban Indonesia. The findings offer a comprehensive view of both the personal and structural dimensions that shape breastfeeding outcomes.

Internally, mothers’ knowledge, self-efficacy, and breastfeeding practices played a pivotal role. Participants with adequate understanding of breastfeeding benefits and techniques were more likely to initiate and sustain exclusive breastfeeding, particularly when paired with strong self-belief. However, even informed mothers reported a significant gap between theory and real-world practice. Many experienced emotional strain, physical fatigue, and uncertainty—especially during periods of low milk supply—making self-efficacy a critical protective factor [13, 14]. Those with previous experience, peer support, or access to modern tools like hands-free pumps demonstrated greater confidence and perseverance [15].

External factors further shaped these outcomes. Supportive environments—particularly from spouses, family, coworkers, and healthcare providers—greatly facilitated mothers’ efforts. Spouses who shared household duties or provided emotional reassurance were instrumental [16, 17]. Peers who normalized pumping breaks, supervisors who showed understanding, and caregivers who reliably managed expressed milk all acted as practical enablers. A unique contribution of this study lies in highlighting the role of domestic helpers and extended family members as caregivers in Indonesia. Due to short maternity leave policies, many working mothers relied heavily on babysitters, household assistants, or grandmothers to support infant feeding. While this arrangement provided essential practical assistance, it also created challenges when caregivers were inadequately trained in handling expressed breast milk or when inconsistent feeding practices occurred. This sociocultural context is less documented in global breastfeeding literature, where formal childcare systems are more common. By drawing attention to the dual role of domestic helpers as both facilitators and potential barriers, this study contributes novel insight into how culturally specific caregiving arrangements intersect with exclusive breastfeeding practices [18–20].

Institutionally, barriers emerged in the form of inadequate lactation facilities, lack of formal pumping breaks, and short maternity leave. Despite legal protections in Indonesia, the implementation remains weak, requiring mothers to negotiate individually for space and time [9, 11]. These findings mirror global trends in other developing countries such as Nigeria, Vietnam, where policy enforcement is limited and workplace accommodations are insufficient [21]. In contrast, nations with structured breastfeeding policies—such as the U.S. and Canada—demonstrate how workplace accommodation and legal mandates, including the Break Time for Nursing Mothers provision under the Affordable Care Act, can foster more consistent support [22, 23].

Maternity leave and workplace conditions significantly influence exclusive breastfeeding practices in Southeast Asia. In the Philippines, policies like the 105-Day Expanded Maternity Leave Law aim to support breastfeeding, yet gaps in implementation hinder their effectiveness, such as inconsistent access and inadequate workplace support for lactation [24]. In Indonesia, a study revealed that only 62% of working mothers in Aceh Jaya practiced exclusive breastfeeding, with factors like knowledge, attitudes, and workplace support playing critical roles [25]. Similarly, in Thailand, mothers expressed a need for designated breastfeeding areas at work, highlighting the importance of supportive environments for lactating employees [26]. Research across 38 low- and middle-income countries indicates that extending paid maternity leave correlates with improved breastfeeding rates, suggesting that longer leave can mitigate barriers faced by working mothers [27]. Overall, enhancing maternity leave policies and workplace conditions is essential for promoting exclusive breastfeeding in the region.

Cultural attitudes also influenced mothers’ experiences. Societal discomfort with public or workplace breastfeeding added psychological barriers, while persistent traditions—such as prelacteal feeding or misinformation from older generations—complicated maternal efforts [28]. The role of domestic helpers, a unique aspect of the Indonesian context, added another layer: while some mothers found their support invaluable, others reported stress due to mishandling or dishonesty, highlighting the need for caregiver training. These patterns are consistent with global literature. In rural Pakistan and Kyrgyzstan, cultural expectations and pressure to supplement breast milk reduce exclusive breastfeeding [19].

By contrast, working mothers in Indonesia remain dependent on employer discretion or informal negotiation to access pumping time and facilities. This places a disproportionate burden on women and reinforces the importance of workplace-level social support, such as understanding supervisors and empathetic colleagues, to sustain exclusive breastfeeding in professional environments. These disparities underscore the urgent need for clear, enforceable breastfeeding policies in Indonesia, including protected pumping breaks and breastfeeding-friendly infrastructure.

To address the negative stigma surrounding breastfeeding among working mothers in patriarchal societies, a multifaceted approach is needed—one that combines policy reform, workplace accommodations, and broader sociocultural change. First, implementing comprehensive maternity protection policies, such as extended paid maternity leave and dedicated lactation facilities, has been shown to improve both maternal and infant health outcomes and workplace productivity [21, 29]. In countries like Ethiopia, where legal protections remain limited, alignment with international standards such as those recommended by the International Labour Organization (ILO) is essential for ensuring breastfeeding rights and promoting optimal practices [28].

In line with ILO Convention No. 183, extending paid maternity leave to at least six months is considered the global standard to support exclusive breastfeeding. However, in Indonesia, implementing this reform remains challenging, as employers often perceive extended leave as a financial and productivity burden, which may unintentionally reinforce gender-based hiring discrimination. Therefore, while advocating for gradual extension of maternity leave, it is equally important to establish employer accountability mechanisms—such as government monitoring and enforcement of workplace lactation rooms and breastfeeding breaks—that can be implemented in the short term. In addition, integrating lactation counseling into workplace health services provides a feasible and cost-effective strategy to support both mothers and caregivers in sustaining breastfeeding [24, 30]. 

Moreover, structural barriers—such as the aggressive marketing of commercial formula—must be addressed, alongside efforts to enhance the capacity of healthcare providers to deliver informed, consistent breastfeeding support [31]. Advocacy initiatives should also prioritize building strong partnerships with stakeholders to foster breastfeeding-friendly policies and environments [21]. On the cultural front, confronting hostile sexism and societal discomfort around public breastfeeding is vital. This can be achieved through public information campaigns that normalize breastfeeding, promote maternal autonomy, and reshape societal narratives about breastfeeding as a private or shameful act [32].

Overall, this study confirms that exclusive breastfeeding among working mothers is shaped by complex, overlapping influences. To close the gap between mothers’ intentions and lived realities, interventions must be holistic—improving individual confidence, workplace conditions, healthcare guidance, and public awareness. These findings provide valuable insight for designing targeted policies and programs that support breastfeeding as a shared societal responsibility rather than an individual burden.

Future interventions to support exclusive breastfeeding among working mothers should include comprehensive workplace policies aligned with international standards, enhanced prenatal breastfeeding education, and targeted support for frontliner professions. Community awareness campaigns to normalize breastfeeding and encourage family involvement—particularly partner support—are also recommended to shift cultural narratives and reduce stigma.

### Limitation

This study was limited by its relatively small sample size and focus on urban settings in Java, which may not fully represent the experiences of all working mothers in Indonesia, particularly those in rural areas or informal employment. Additionally, data were self-reported, which may introduce recall or social desirability bias. Future studies should purposively include mothers who discontinued exclusive breastfeeding (EBF) earlier to better understand barriers leading to cessation.

## Conclusion

This study explored how internal and external factors shape the exclusive breastfeeding experiences of formally employed mothers in urban Indonesia. The findings reveal that internal factors—such as maternal knowledge, self-efficacy, and adaptive breastfeeding practices—serve as key enablers but are often challenged by fatigue, stress, and the gap between theoretical knowledge and daily practice. External factors, including family and peer support, workplace policies, and cultural attitudes, can either facilitate or hinder breastfeeding depending on their consistency and responsiveness.

Barriers such as limited lactation facilities, inflexible work schedules, social stigma, and inconsistent caregiver support emerged as significant challenges. Conversely, strong partner involvement, empathetic supervisors, accessible lactation rooms, and peer solidarity were powerful facilitators of exclusive breastfeeding. By identifying these barriers and facilitators through the lived experiences of working mothers, this study offers critical insights for designing policies and interventions that are both culturally sensitive and structurally supportive, helping bridge the gap between intention and practice in achieving exclusive breastfeeding.

## Supplementary Information

Supplementary Material 1.

## Data Availability

The datasets generated and/or analyzed during the current study are not publicly available due to privacy and ethical restrictions, but are available from the corresponding author on reasonable request.
