# Supplementary material for: Barriers and facilitators to exclusive breastfeeding among formally employed mothers in urban Indonesia
Source: BMC Public Health. 2025 Nov 12;25:3925. doi: 10.1186/s12889-025-25214-6 (PMC12613390; doi:10.1186/s12889-025-25214-6)
Supplement: Supplementary file 1 — Supplementary Material 1. [file 12889_2025_25214_MOESM1_ESM.docx]

Supplementary File 1

**IN-DEPTH INTERVIEW GUIDE**

**General Data to Be Recorded for Each Interview:**

- a) Interviewer Name:
- b) Note Taker Name:
- c) Date of Interview:
- d) Respondent's Full Name:
- e) Respondent's Occupation:
- f) Respondent's Phone Number:

**INTERVIEW OPENING STAGE**

1. Express appreciation to the respondent for their willingness to participate in the interview.
2. Introduce yourself and explain the purpose and topic of the interview.
3. Inform the respondent that they are free to share their opinions, experiences, and expectations related to the topic.
4. Record and take notes of the entire conversation. An audio recording device may be used to assist the note-taking process.
5. If the respondent has limited time, an alternative schedule can be arranged based on mutual availability.

**INTERVIEW IMPLEMENTATION STAGE**

The interview will be conducted directly by the researcher. The interviewer will ask questions aligned with the research topic and objectives, focusing on **internal and external factors influencing exclusive breastfeeding**. The main themes of the questions are as follows:

**1. Internal Factors**

**1.1 Maternal Characteristics:**

- 1.1.1 Maternal Age
- 1.1.2 Education Level
- 1.1.3 Occupation: Type of Job, Working Hours, Duration of Employment
- 1.1.4 Income Level
- 1.1.5 Marital Status
- 1.1.6 Parity

**1.2 Knowledge:**

- 1.2.1 Breast Milk Composition
- 1.2.2 Benefits of Breast Milk
- 1.2.3 Duration of Exclusive Breastfeeding

**1.3 Stress Management:**

- 1.3.1 Coping Mechanisms
- 1.3.2 Frequency of Stress

**1.4 Self-Efficacy:**

- 1.4.1 Confidence
- 1.4.2 Motivation
- 1.4.3 Ability to Overcome Challenges

**2. External Factors**

**2.1 Social Support:**

- 2.1.1 Family Support: Husband, Parents, In-laws
- 2.1.2 Coworker Support
- 2.1.3 Supervisor Support

**2.2 Workplace Factors:**

- 2.2.1 Availability of Lactation Room Facilities
- 2.2.2 Flexibility of Time for Expressing Breast Milk
- 2.2.3 Maternity Leave Policy

**INTERVIEW CLOSING STAGE**

Thank the respondent and ask whether they are willing to be contacted again for further clarification if needed.
